# Cortical Depth Dependence of the Diffusion Anisotropy in the Human Cortical Gray Matter *In Vivo*

**DOI:** 10.1371/journal.pone.0091424

**Published:** 2014-03-07

**Authors:** Trong-Kha Truong, Arnaud Guidon, Allen W. Song

**Affiliations:** Brain Imaging and Analysis Center, Duke University, Durham, North Carolina, United States of America; University of Alberta, Canada

## Abstract

Diffusion tensor imaging (DTI) is typically used to study white matter fiber pathways, but may also be valuable to assess the microstructure of cortical gray matter. Although cortical diffusion anisotropy has previously been observed in vivo, its cortical depth dependence has mostly been examined in high-resolution ex vivo studies. This study thus aims to investigate the cortical depth dependence of the diffusion anisotropy in the human cortex in vivo on a clinical 3 T scanner. Specifically, a novel multishot constant-density spiral DTI technique with inherent correction of motion-induced phase errors was used to achieve a high spatial resolution (0.625×0.625×3 mm) and high spatial fidelity with no scan time penalty. The results show: (i) a diffusion anisotropy in the cortical gray matter, with a primarily radial diffusion orientation, as observed in previous ex vivo and in vivo studies, and (ii) a cortical depth dependence of the fractional anisotropy, with consistently higher values in the middle cortical lamina than in the deep and superficial cortical laminae, as observed in previous ex vivo studies. These results, which are consistent across subjects, demonstrate the feasibility of this technique for investigating the cortical depth dependence of the diffusion anisotropy in the human cortex in vivo.

## Introduction

Diffusion tensor imaging (DTI) has traditionally been used to study fiber pathways in the white matter (WM), but its application for the investigation of diffusion anisotropy in the human cortical gray matter (GM) may also be highly valuable in both basic and clinical neurosciences. For example, it could potentially be used to characterize the cortical cyto- and myeloarchitecture noninvasively or to detect cortical abnormalities in various neurological disorders.

Cortical diffusion anisotropy has previously been observed in a number of DTI studies performed in the developing brain, including in the animal brain ex vivo [1]–[3], in the animal brain in vivo [4], [5], in the human brain ex vivo [6], [7], and in the human brain in vivo [8]–[11]. More recently, it has also been observed in high-resolution DTI studies performed in the adult brain, including in the macaque brain in vivo [12], in the human brain ex vivo [12]–[14], and in the human brain in vivo [12], [15]–[19].

However, since the cortex is only a few millimeters thick, investigating the cortical depth dependence of the diffusion anisotropy requires a much higher spatial resolution than the typical 2–3 mm isotropic resolution used for human in vivo applications. As such, it has so far mostly been performed in animal or human ex vivo brain samples [1], [20]–[26]. Such studies can afford very long scan times (i.e., hours or even days) and take advantage of small-bore MRI scanners with ultra-high field strengths and strong gradients to achieve a sufficiently high spatial resolution and signal-to-noise ratio (SNR). They are also useful in that they can be validated with histology in the same samples. However, the generalization of the findings may be limited by differences across species, subjects, or brain regions and by alterations to tissue properties due to death or tissue fixation. The ability to investigate the cortical depth dependence of the diffusion anisotropy in the human cortex in vivo and hence to obtain subject-specific, whole-brain results, preferably on a clinical (e.g., 3 T) scanner and in a general population, would therefore be extremely valuable.

For human in vivo studies, DTI is typically performed with single-shot echo-planar imaging, which is generally limited by a low spatial resolution, a low SNR, and a high vulnerability to geometric distortions. Multishot spiral DTI, on the other hand, can achieve a higher spatial resolution and SNR, but is more challenging because subject motion causes phase errors among different shots, leading to signal loss and aliasing artifacts in the reconstructed images as well as subsequent errors in the resulting DTI metrics and fiber tracts.

These motion-induced phase errors can be estimated and corrected by using a variable-density spiral trajectory [27], [28] or a navigator echo [29], however at the cost of a longer scan time, which limits the spatial and/or angular resolution. These errors can also be corrected by using an iterative phase correction method [30], which does not require any additional data acquisition, but suffers from long computation times. To address these issues, we recently proposed an alternative method that can inherently and more efficiently correct for phase errors caused by both rigid and nonrigid (e.g., pulsatile) motion, without increasing the scan time or reducing the SNR [18].

In the present study, we apply this novel multishot constant-density spiral DTI technique with inherent correction of motion-induced phase errors to achieve a high spatial resolution and spatial fidelity and to demonstrate its feasibility for investigating the cortical depth dependence of the diffusion anisotropy in the human cortex in vivo at 3 T.

## Methods

### Data Acquisition

We studied three healthy adult volunteers on a 3 T MR750 MRI scanner (GE Healthcare, Milwaukee, WI) equipped with a 32-channel phased-array head coil (Nova Medical, Wilmington, MA) and a gradient system with 50 mT/m maximum amplitude and 200 T/m/s maximum slew rate. All subjects provided written informed consent to participate in this study under a protocol approved by the Duke University Health System Institutional Review Board. Foam padding was used to restrain the head within the coil and high-order shimming was performed to reduce the global B_0_ inhomogeneity.

Multishot spiral DTI data were acquired in ten contiguous axial slices at the top of the brain with a single spin-echo spiral sequence and with repetition time = 2 s, echo time = 51 ms, field-of-view = 16×16 cm, matrix size = 256×256 (for both the acquisition and reconstruction), in-plane resolution = 625×625 µm, slice thickness = 3 mm, b-factor = 800 s/mm^2^, number of diffusion-weighting directions = 60, number of baseline (b = 0) images = 7, number of averages = 1, and scan time = 13.6 min. A spatial-spectral pulse was used for fat suppression and the k-space trajectory was a six-shot constant-density spiral-out trajectory with 10,892 data points per interleaf and a readout duration of 44 ms.

Sagittal T_1_-weighted anatomical images of the whole brain were also acquired with a 3D inversion-prepared spoiled gradient-echo sequence and with repetition time = 8.1 ms, echo time = 3.2 ms, inversion time = 450 ms, flip angle = 12^o^, voxel size = 1×1×1 mm (interpolated to 625×625×625 µm), acceleration factor = 1.83, and scan time = 5 min. In addition, a B_0_ map was acquired in vivo to correct for blurring artifacts caused by susceptibility effects near air/tissue interfaces, whereas B_0_ maps were acquired on a phantom with the same diffusion-weighting gradients as in the DTI scan to correct for blurring artifacts caused by eddy currents [31]. The whole experiment was performed twice on subject #1 (on two different days) to assess the reproducibility of the results and once on subjects #2 and #3 to assess the consistency of the results across different subjects.

### Data Reconstruction and Analysis

Motion-induced phase errors, signal loss, and aliasing artifacts in the multishot spiral DTI data were inherently corrected as described in [18]. Specifically, the central k-space data of each shot were first reconstructed by using a sensitivity encoding (SENSE) reconstruction algorithm [32] to generate low-resolution phase images, which were smoothed to yield estimates of the motion-induced phase errors. These phase errors were then corrected by using an iterative conjugate gradient algorithm [27].

Blurring artifacts due to susceptibility effects and eddy currents were also corrected by using the acquired B_0_ maps, as described in [31]. The DTI images were then registered to the anatomical images with FMRIB’s Linear Image Registration Tool (FLIRT) [33] before derivation of DTI metrics, including the axial and radial diffusivity, fractional anisotropy (FA), directionally-encoded color (DEC), and principal eigenvector.

To investigate the cortical depth dependence of the diffusion anisotropy, 13 surfaces evenly spaced along the cortical depth in increments of 10% of the cortical thickness were generated from the anatomical images with FreeSurfer [34]. These surfaces included the pial surface and the GM/WM interface (at a cortical depth of 0% and 100%), nine intermediate surfaces in cortical GM (at a cortical depth ranging from 10% to 90%), and two additional surfaces in the adjacent cerebrospinal fluid (CSF) or WM (at a cortical depth of −10% and 110%).

Cortical profiles of the FA as a function of the cortical depth were computed by averaging the FA within each of these surfaces. To minimize partial volume effects along the slice direction (since the DTI slice thickness was larger than the in-plane resolution), the normal vectors to the GM/WM interface were generated from the anatomical images with FreeSurfer. Since the anatomical images had a higher spatial resolution along z than the DTI data, normal vectors were generated at multiple locations along z within each DTI voxel. Then, only voxels for which the normal vectors at all of these locations were within ±20^o^ of the axial plane (i.e., voxels for which the GM/WM interface was approximately flat and orthogonal to the axial plane) were used to compute the cortical profiles. Furthermore, to assess the consistency of the results across different cortical regions, separate profiles were computed in different regions-of-interest (ROIs) placed in various anatomical locations within the acquired slices.

The same method was also used to compute cortical profiles of the axial diffusivity, radial diffusivity, and radiality index, which is defined as the absolute value of the dot product between the principal eigenvector of the diffusion tensor and the normal vector to the cortical surface (1 = radial, 0 = tangential) [12]. All data reconstruction and analysis were performed in Matlab (The MathWorks, Natick, MA) unless stated otherwise.

Note that for a 625 µm in-plane resolution, there are at most a few voxels across the cortex at any given location along the cortical ribbon (e.g., about 5 voxels for a cortical thickness of 3 mm). However, using intermediate surfaces with a finer spacing than the in-plane resolution allows different cortical depths to be sampled at different locations along the cortical ribbon within an ROI, resulting in more uniformly sampled cortical profiles [12].

## Results

**Figure 1:**
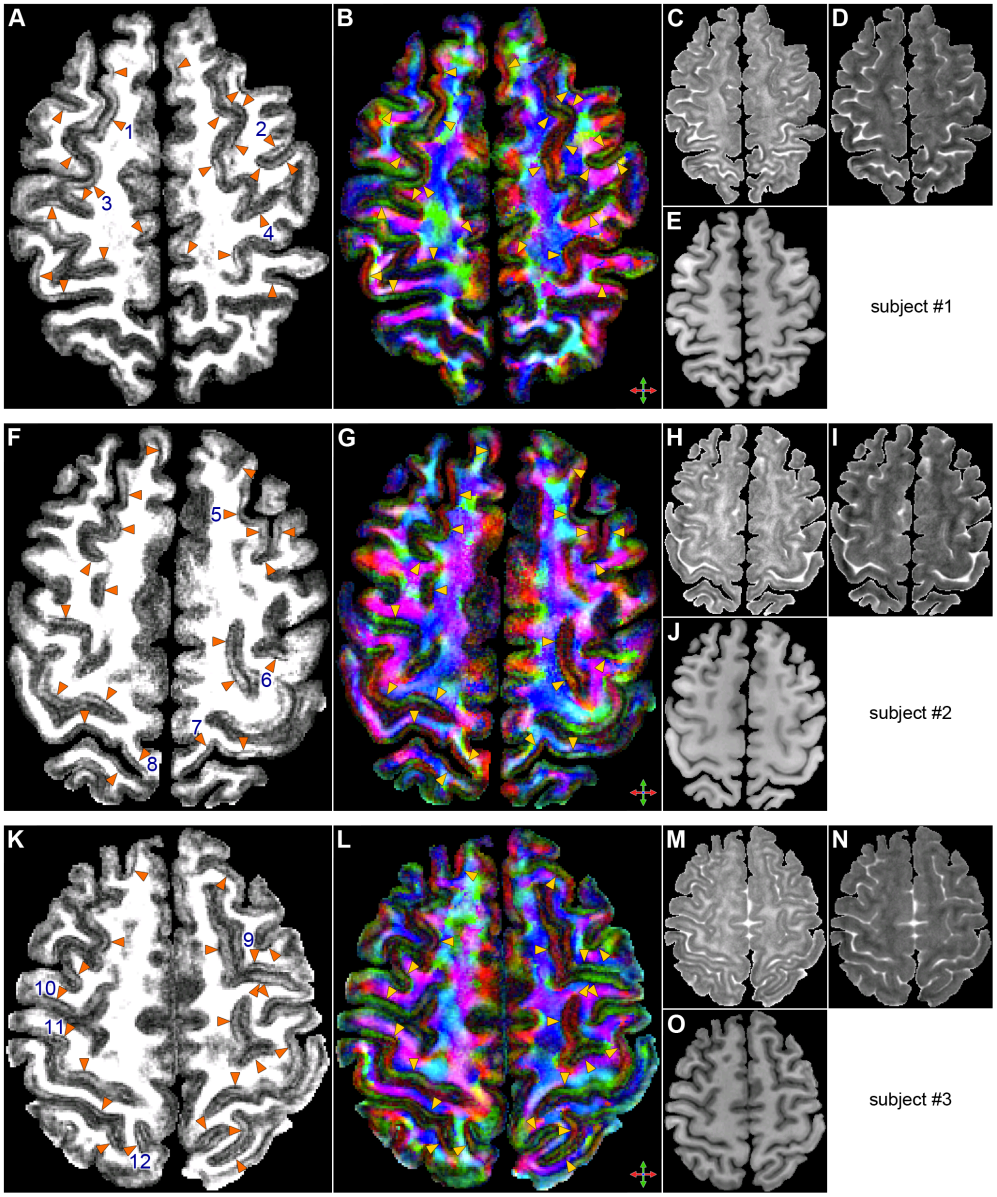
DTI results. Maps of the FA (A,F,K), DEC (B,G,L), axial diffusivity (C,H,M), and radial diffusivity (D,I,N) and T_1_-weighted anatomical images (E,J,O) for three different subjects. The FA and DEC maps are scaled from 0 to 0.3 to highlight the FA variations within the cortical GM and the diffusivity maps are scaled from 0 to 2×10^−3^ mm^2^/s. The arrowheads in (A,B,F,G,K,L) show a band of low FA in the deep cortical lamina. The numbers in (A,F,K) refer to the ROIs used in Figs. 5 and 7.

**Figure 2:**
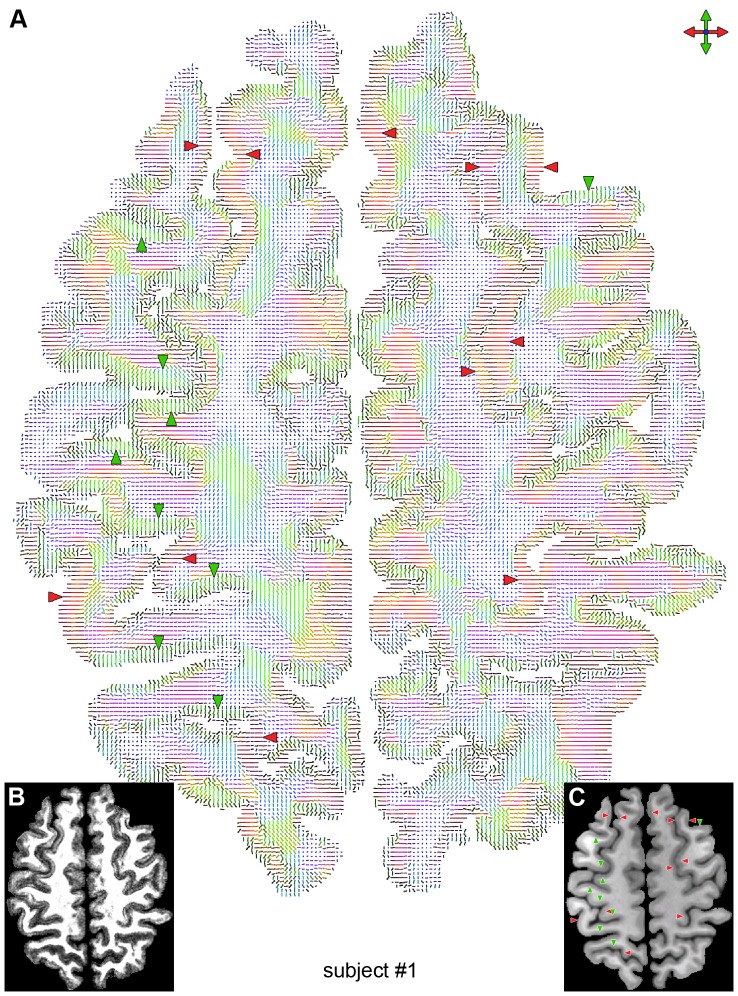
Principal diffusion direction for subject #1. Color-coded map of the principal eigenvector (A), FA map (B), and T_1_-weighted anatomical image (C) in the same slice as that shown in Figs. 1A–E. The green and red arrowheads show cortical GM regions with a primarily radial principal diffusion direction.

**Figure 3:**
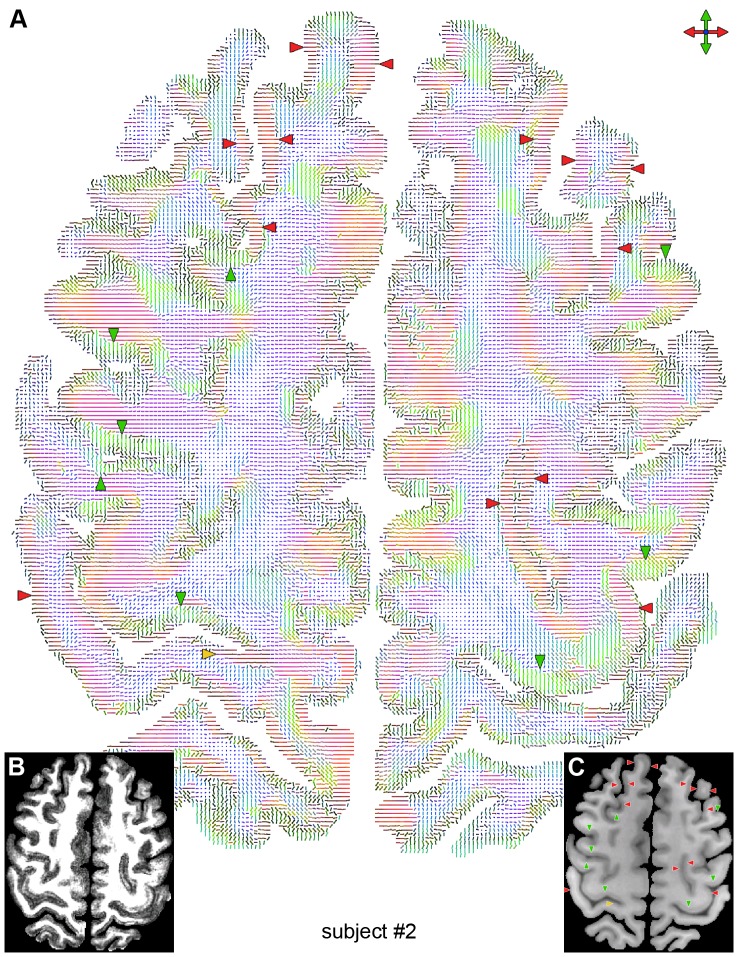
Principal diffusion direction for subject #2. Color-coded map of the principal eigenvector (A), FA map (B), and T_1_-weighted anatomical image (C) in the same slice as that shown in Figs. 1F–J. The green and red arrowheads show cortical GM regions with a primarily radial principal diffusion direction, whereas the yellow arrowhead shows a cortical GM region with a primarily tangential principal diffusion direction.

**Figure 4:**
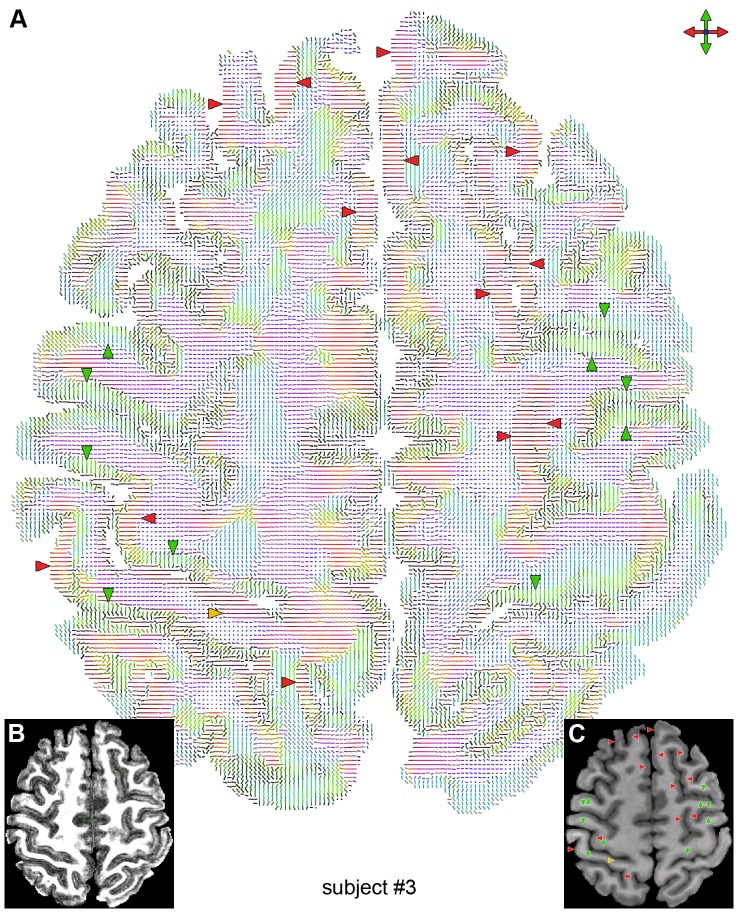
Principal diffusion direction for subject #3. Color-coded map of the principal eigenvector (A), FA map (B), and T_1_-weighted anatomical image (C) in the same slice as that shown in Figs. 1K–O. The green and red arrowheads show cortical GM regions with a primarily radial principal diffusion direction, whereas the yellow arrowhead shows a cortical GM region with a primarily tangential principal diffusion direction.

The high-quality, high-resolution multishot spiral DTI results obtained with our method reveal features typically not seen in conventional DTI data acquired in vivo at 3 T. In particular, the FA (Figs. 1A,F,K) and DEC (Figs. 1B,G,L) maps clearly show a diffusion anisotropy in the cortical GM, whereas the color-coded maps of the principal eigenvector (Figs. 2A, 3A, 4A) show that most of the cortical GM regions have a primarily radial diffusion orientation (i.e., orthogonal to the cortical surface). For example, cortical GM regions oriented along the right/left direction generally have a principal eigenvector along the anterior/posterior direction (green arrowheads in Figs. 2A, 3A, 4A), while cortical GM regions oriented along the anterior/posterior direction generally have a principal eigenvector along the right/left direction (red arrowheads in Figs. 2A, 3A, 4A). There are, however, also a few cortical GM regions that have a primarily tangential diffusion orientation (yellow arrowheads in Figs. 3A, 4A).

Although cortical GM appears uniform in the T_1_-weighted anatomical images (Figs. 1E,J,O), a striking feature in the FA (Figs. 1A,F,K) and DEC (Figs. 1B,G,L) maps is the clear cortical depth dependence of the diffusion anisotropy throughout the brain, characterized by a band of low FA in the deep cortical lamina adjacent to the GM/WM interface (arrowheads in Figs. 1A,B,F,G,K,L) and a band of higher FA in the middle cortical lamina. (In this work, the superficial, middle, and deep cortical laminae are loosely defined as the regions with a cortical depth of 0–33%, 33–67%, and 67–100%, respectively, but do not refer to individual cortical layers, which are not evenly spaced along the cortical depth.).

**Figure 5:**
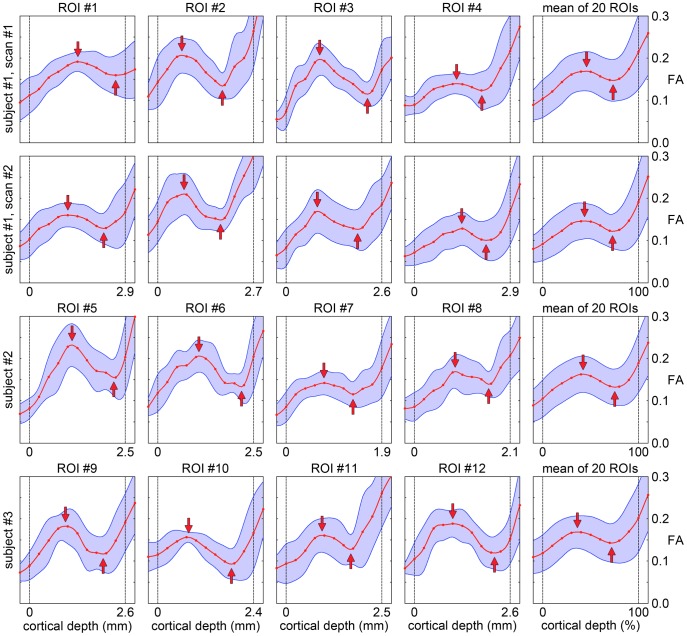
Cortical profiles of the FA. FA (mean ± standard deviation) as a function of the cortical depth. The solid lines were computed with cubic spline interpolation. The dashed lines denote the pial surface and the GM/WM interface at a cortical depth of 0% and 100%. The first four columns show the cortical profiles in the representative ROIs shown in Figs. 1A,F,K, whereas the last column shows the mean cortical profiles averaged over 20 ROIs. The first two rows show results from identical experiments performed on the same subject on two different days. The arrows show a local maximum in the middle cortical lamina and a local minimum in the deep cortical lamina.

This cortical depth dependence can be better seen in the cortical profiles of the FA (Fig. 5), which consistently show a local maximum in the middle cortical lamina, at a cortical depth of (40±10)%, and a local minimum in the deep cortical lamina, at a cortical depth of (74±11)%, in all ROIs (arrows in Fig. 5). Although there is some variability across different ROIs, cortical profiles measured on the same subject on two different days show a remarkably good agreement in all ROIs (first two rows in Fig. 5). Furthermore, the mean cortical profiles averaged over 20 ROIs are highly consistent across all subjects (last column in Fig. 5).

In theory, the variability across different ROIs may be partly due to the FA dependence on the underlying cyto- and myeloarchitecture, which can vary across different cortical regions, but may also be partly due to partial volume effects or misregistration between the DTI data and the surfaces generated with FreeSurfer. However, the high reproducibility of the results obtained on the same subject on two different days suggests that the first contribution seems to be dominant, although some of the variability could still be due to systematic errors affecting both experiments.

Partial volume averaging between GM and the adjacent CSF or WM, which generally have a lower or higher FA, respectively, can potentially bias the FA measurements. Specifically, partial volume averaging between GM and CSF may result in a lower FA near the pial surface, whereas partial volume averaging between GM and WM may result in a higher or lower FA near the GM/WM interface, depending on whether the diffusion orientation is similar or different in the GM and WM. However, partial volume effects alone cannot explain why the cortical profiles of the FA consistently show a local maximum in the middle cortical lamina and a local minimum in the deep cortical lamina rather than at the GM/WM interface. Partial volume effects may only affect the location of these peaks, but since the cortical profiles were only computed from voxels for which the GM/WM interface was approximately flat and orthogonal to the slice, such effects would at most affect a 625-µm thick layer of cortex beyond the pial surface or the GM/WM interface, which would typically not include the middle cortical lamina.

**Figure 6:**
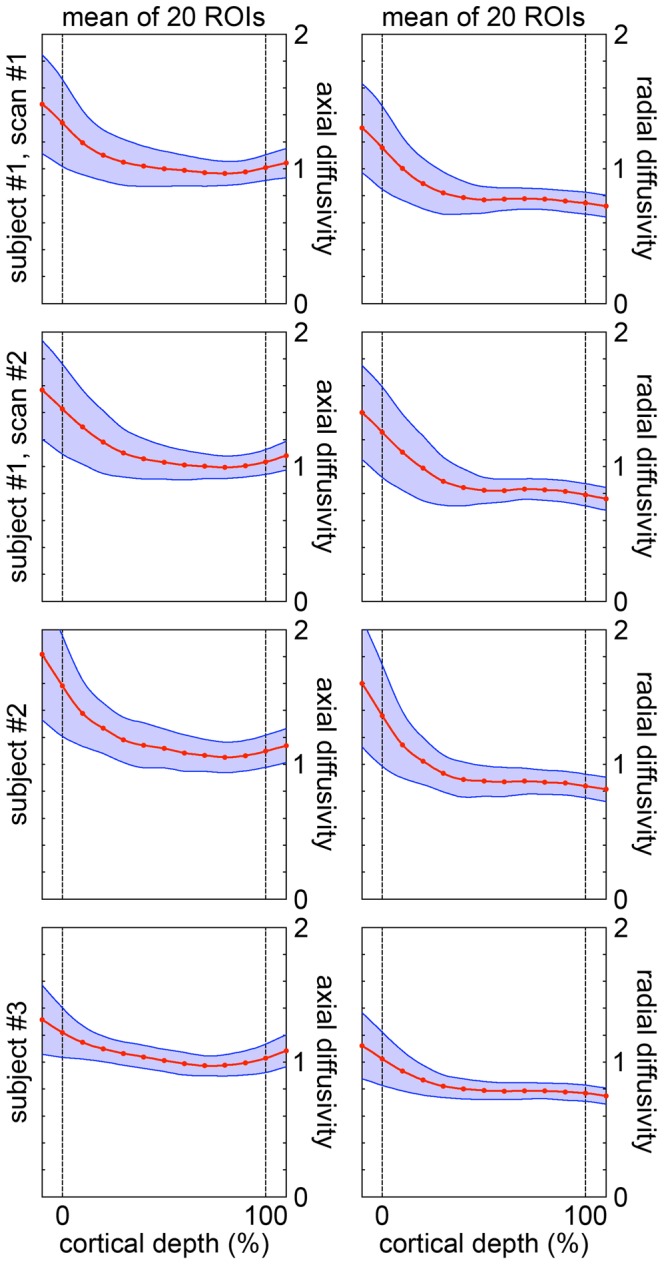
Cortical profiles of the diffusivity. Axial and radial diffusivity (mean ± standard deviation in 10^−3^ mm^2^/s) as a function of the cortical depth. The solid lines were computed with cubic spline interpolation. The dashed lines denote the pial surface and the GM/WM interface at a cortical depth of 0% and 100%. Both columns show the mean cortical profiles averaged over 20 ROIs. The first two rows show results from identical experiments performed on the same subject on two different days.

**Figure 7:**
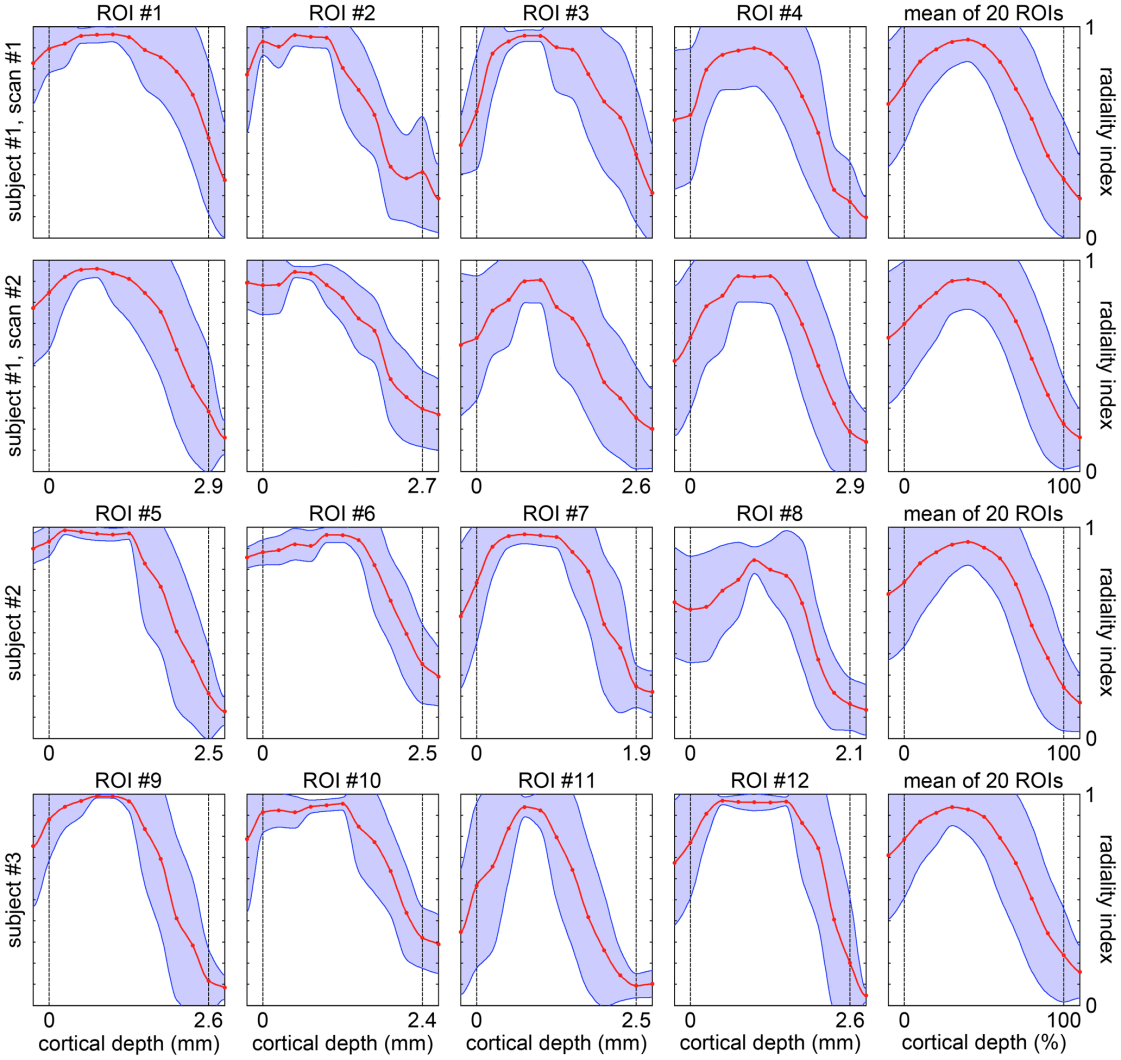
Cortical profiles of the radiality index. Radiality index (mean ± standard deviation; 1 = radial, 0 = tangential) as a function of the cortical depth. The solid lines were computed with cubic spline interpolation. The dashed lines denote the pial surface and the GM/WM interface at a cortical depth of 0% and 100%. The first four columns show the cortical profiles in the representative ROIs shown in Figs. 1A,F,K, whereas the last column shows the mean cortical profiles averaged over 20 ROIs. The first two rows show results from identical experiments performed on the same subject on two different days.

The cortical profiles of the axial and radial diffusivity (Fig. 6) do not show any notable local maximum or minimum, whereas the cortical profiles of the radiality index (Fig. 7) show a maximum in the middle cortical lamina, at a cortical depth of (35±12)%, in nearly all ROIs.

## Discussion

The results of this study, performed in the human brain in vivo at 3 T, show: (i) a clear diffusion anisotropy in the cortical GM, with a primarily radial diffusion orientation, and (ii) a cortical depth dependence of the FA, with consistently higher values in the middle cortical lamina than in the deep and superficial cortical laminae.

Such a radial diffusion orientation in cortical GM has initially been observed in DTI studies performed in the developing brain [2], [5], [6], [8]–[10], owing to its high cortical diffusion anisotropy, which is then markedly reduced as microstructural changes occur during brain maturation. More recently, radial diffusion anisotropy in the cortex has also been observed in high-resolution DTI studies performed in the adult brain, including in the pig brain ex vivo [20], in the macaque brain in vivo [12], in the human brain ex vivo [12]–[14], and in the human brain in vivo [12], [15]–[19]. In the latter case, however, most of the results were obtained with a reduced field-of-view, a 7 T scanner, long scan times (about 1 hour), and/or a customized 100 mT/m gradient coil.

Some of these in vivo studies, performed with a 1–1.5 mm isotropic resolution, also showed a regional dependence of the principal diffusion direction, specifically a radial diffusion orientation in the primary motor cortex (M1) and a tangential diffusion orientation in the primary somatosensory cortex (S1) [12], [35]. Our results show that most of the cortical GM regions, including M1, have a primarily radial diffusion orientation, but there are also a few regions, including parts of S1 (yellow arrowheads in Figs. 3A, 4A), that have a primarily tangential diffusion orientation. However, the tangential orientation in S1 is not consistently seen in both hemispheres and in all subjects, possibly because of the anisotropic voxel size. Additional studies, ideally performed with an isotropic voxel size, are needed to further investigate the regional dependence of the principal diffusion direction.

The band of low FA seen in the deep cortical lamina (arrowheads in Figs. 1A,B,F,G,K,L) can also be discerned in other high-resolution DTI studies, including in the cat brain in vivo [36], in the macaque brain in vivo [12], in the human brain ex vivo [13], [14], and, to a lesser extent, in the human brain in vivo [12], [18].

More quantitatively, the cortical profiles of the FA obtained in our in vivo study (Fig. 5) are consistent with those obtained in two recent ex vivo DTI studies performed in the human M1 and S1 [24] and in the human secondary visual cortex (V2) [26] with a 242–300 µm isotropic resolution, both of which also showed a maximum diffusion anisotropy in the middle cortical lamina, at a cortical depth of 33% (M1), 44% (S1), and 35–43% (V2). (Note that the first study did not show cortical profiles of the FA, but of the ratio of the two largest peaks of the fiber orientation distribution function, which is a different way to characterize the diffusion anisotropy.).

Furthermore, variations in the cortical profiles across different regions within the same brain samples were also observed in these studies, specifically between M1 and S1 [24] and, to a larger extent, between the primary visual cortex (V1) and V2 [26]. In V1 (which was not included in our data), a local minimum rather than maximum of the FA was actually observed in the middle cortical lamina at a cortical depth close to that of the stria of Gennari in layer IVb, which contains both radial and tangential fibers.

Previous ex vivo DTI studies performed in V1, M1, and S1 with a 188×188×376 µm or 242 µm isotropic resolution [21], [23] also showed a cortical depth dependence of the principal diffusion direction, specifically a radial diffusion orientation in the middle cortical lamina and a tangential diffusion orientation in the deep and superficial cortical laminae. An in vivo DTI study performed with a 1 mm isotropic resolution [12] found a similar dependence in M1, but not in S1. In addition, the ex vivo DTI study performed in V2 with a 300 µm isotropic resolution [26] further showed a correlation between the fiber orientation distributions and the cortical profiles of the FA. The middle cortical lamina had a primarily radial diffusion orientation, corresponding to the FA maximum in the cortical profiles, whereas the deep cortical lamina contained both radial and tangential diffusion orientations, corresponding to the FA minimum in the cortical profiles.

The cortical profiles of the FA (Fig. 5) and radiality index (Fig. 7) obtained in our in vivo study are consistent with these findings and with each other. In the middle cortical lamina, the principal diffusion direction is primarily radial and both the FA and radiality index reach a maximum at a cortical depth of (40±10)% and (35±12)%, respectively. In the deep cortical lamina, the principal diffusion direction gradually changes from radial to tangential and the FA reaches a minimum while the radiality index crosses 0.707 (corresponding to a 45^o^ angle between the principal eigenvector and the normal vector to the cortical surface) at a cortical depth of (74±11)% and (71±9)%, respectively.

While the present study was not designed to demonstrate a cortical depth dependence of the diffusion orientations on a voxel-by-voxel basis, as shown in previous ex vivo DTI studies performed with a higher resolution (242–300 µm isotropic) [22], [26], it did, however, consistently show a cortical depth dependence of the diffusion anisotropy, both in the FA maps and in the cortical profiles of the FA averaged within different ROIs. Importantly, these cortical profiles obtained in vivo on a clinical 3 T scanner are in good agreement with those obtained ex vivo on small-bore scanners with much higher field strengths (9.4–11.7 T), stronger gradients (600–1500 mT/m), and longer scan times (e.g., 14 hours) [24], [26]. These findings bridge the gap and illustrate the complementarity between ex vivo studies, which can be performed at a very high resolution and be validated with histology [22], [37], and in vivo studies, which can provide whole-brain, subject-specific results and potentially lead to a much broader range of applications.

This proof-of-concept study only focused on ten axial slices at the top of the brain and used a larger slice thickness than the in-plane resolution. However, special care was taken to minimize partial volume effects along the slice direction by restricting the data analysis to voxels for which the GM/WM interface was approximately flat and orthogonal to the slice. As a result, it was not possible to place ROIs in all anatomical locations throughout the brain, either because they were not included in the acquired slices or because they did not have a GM/WM interface that was approximately flat and orthogonal to the slice. It was therefore also not possible to place all of the ROIs in similar locations across all subjects, because of individual variations in the orientation of the cortical surface (although ROIs #1–4 were placed in similar locations for both scans on subject #1). Nevertheless, ROIs were placed in as many locations as possible within the acquired slices and cortical profiles of the FA were computed both in individual ROIs and averaged over all ROIs to demonstrate that the results are consistent across ROIs and subjects.

A more systematic investigation of the variability of the results across different cortical regions and of their consistency across different subjects would ideally require a whole-brain coverage and an isotropic voxel size, which would allow ROIs to be placed in any location throughout the brain and in similar locations across all subjects. However, achieving a sub-millimeter isotropic voxel size requires further technical improvements to increase the SNR (e.g., with 3D acquisition techniques), which are beyond the scope of this work.

## Conclusions

The results of this study show: (i) a clear diffusion anisotropy in the human cortical GM in vivo, with a primarily radial diffusion orientation, as observed in previous ex vivo and in vivo DTI studies, and (ii) a cortical depth dependence of the FA, with consistently higher values in the middle cortical lamina than in the deep and superficial cortical laminae, as observed in previous ex vivo DTI studies. These results, which are consistent across all subjects, demonstrate that our multishot constant-density spiral DTI technique with inherent correction of motion-induced phase errors can be used to study the cortical depth dependence of the diffusion anisotropy in the human cortex in vivo at 3 T, which will be valuable to assess the cortical microstructure noninvasively across a general population.

## References

[1] Kroenke CD, Van Essen DC, Inder TE, Rees S, Bretthorst GL, et al (2007) Microstructural changes of the baboon cerebral cortex during gestational development reflected in magnetic resonance imaging diffusion anisotropy. J Neurosci 27: 12506–12515. 18003829 10.1523/JNEUROSCI.3063-07.2007 PMC4780575

[2] Bock AS, Olavarria JF, Leigland LA, Taber EN, Jespersen SN, et al (2010) Diffusion tensor imaging detects early cerebral cortex abnormalities in neuronal architecture induced by bilateral neonatal enucleation: an experimental model in the ferret. Front Syst Neurosci 4: 149. 21048904 10.3389/fnsys.2010.00149 PMC2971465

[3] Takahashi E, Dai G, Wang R, Ohki K, Rosen GD, et al (2010) Development of cerebral fiber pathways in cats revealed by diffusion spectrum imaging. NeuroImage 49: 1231–1240. 19747553 10.1016/j.neuroimage.2009.09.002 PMC2789885

[4] Thornton JS, Ordidge RJ, Penrice J, Cady EB, Amess PN, et al (1997) Anisotropic water diffusion in white and gray matter of the neonatal piglet brain before and after transient hypoxia-ischaemia. Magn Reson Imaging 15: 433–440. 9223044 10.1016/s0730-725x(96)00378-5

[5] Sizonenko SV, Camm EJ, Garbow JR, Maier SE, Inder TE, et al (2007) Developmental changes and injury induced disruption of the radial organization of the cortex in the immature rat brain revealed by in vivo diffusion tensor MRI. Cereb Cortex 17: 2609–2617. 17259644 10.1093/cercor/bhl168 PMC4780675

[6] Gupta RK, Hasan KM, Trivedi R, Pradhan M, Das V, et al (2005) Diffusion tensor imaging of the developing human cerebrum. J Neurosci Res 81: 172–178. 15931676 10.1002/jnr.20547

[7] Trivedi R, Gupta RK, Husain N, Rathore RK, Saksena S, et al (2009) Region-specific maturation of cerebral cortex in human fetal brain: diffusion tensor imaging and histology. Neuroradiology 51: 567–576. 19421746 10.1007/s00234-009-0533-8

[8] McKinstry RC, Mathur A, Miller JH, Ozcan A, Snyder AZ, et al (2002) Radial organization of developing preterm human cerebral cortex revealed by non-invasive water diffusion anisotropy MRI. Cereb Cortex 12: 1237–1243. 12427675 10.1093/cercor/12.12.1237

[9] Maas LC, Mukherjee P, Carballido-Gamio J, Veeraraghavan S, Miller SP, et al (2004) Early laminar organization of the human cerebrum demonstrated with diffusion tensor imaging in extremely premature infants. NeuroImage 22: 1134–1140. 15219585 10.1016/j.neuroimage.2004.02.035

[10] deIpolyi AR, Mukherjee P, Gill K, Henry RG, Partridge SC, et al (2005) Comparing microstructural and macrostructural development of the cerebral cortex in premature newborns: diffusion tensor imaging versus cortical gyration. NeuroImage 27: 579–586. 15921934 10.1016/j.neuroimage.2005.04.027

[11] Mukherjee P, McKinstry RC (2006) Diffusion tensor imaging and tractography of human brain development. Neuroimaging Clin N Am 16: 19–42. 16543084 10.1016/j.nic.2005.11.004

[12] McNab JA, Polimeni JR, Wang R, Augustinack JC, Fujimoto K, et al (2013) Surface based analysis of diffusion orientation for identifying architectonic domains in the in vivo human cortex. NeuroImage 69: 87–100. 23247190 10.1016/j.neuroimage.2012.11.065 PMC3557597

[13] McNab JA, Jbabdi S, Deoni SC, Douaud G, Behrens TE, et al (2009) High resolution diffusion-weighted imaging in fixed human brain using diffusion-weighted steady state free precession. NeuroImage 46: 775–785. 19344686 10.1016/j.neuroimage.2009.01.008

[14] Miller KL, Stagg CJ, Douaud G, Jbabdi S, Smith SM, et al (2011) Diffusion imaging of whole, post-mortem human brains on a clinical MRI scanner. NeuroImage 57: 167–181. 21473920 10.1016/j.neuroimage.2011.03.070 PMC3115068

[15] Jaermann T, De Zanche N, Staempfli P, Pruessmann KP, Valavanis A, et al (2008) Preliminary experience with visualization of intracortical fibers by focused high-resolution diffusion tensor imaging. AJNR Am J Neuroradiol 29: 146–150. 17947372 10.3174/ajnr.A0742 PMC8119111

[16] Heidemann RM, Anwander A, Feiweier T, Knösche TR, Turner R (2012) k-space and q-space: combining ultra-high spatial and angular resolution in diffusion imaging using ZOOPPA at 7 T. NeuroImage 60: 967–978. 22245337 10.1016/j.neuroimage.2011.12.081

[17] Heidemann RM, Porter DA, Anwander A, Feiweier T, Heberlein K, et al (2010) Diffusion imaging in humans at 7T using readout-segmented EPI and GRAPPA. Magn Reson Med 64: 9–14. 20577977 10.1002/mrm.22480

[18] Truong TK, Guidon A (2014) High-resolution multishot spiral diffusion tensor imaging with inherent correction of motion-induced phase errors. Magn Reson Med 71: 790–796. 23450457 10.1002/mrm.24709 PMC3949176

[19] Sotiropoulos SN, Jbabdi S, Xu J, Andersson JL, Moeller S, et al (2013) Advances in diffusion MRI acquisition and processing in the Human Connectome Project. NeuroImage 80: 125–143. 23702418 10.1016/j.neuroimage.2013.05.057 PMC3720790

[20] Dyrby TB, Baaré WFC, Alexander DC, Jelsing J, Garde E, et al (2011) An ex vivo imaging pipeline for producing high- quality and high-resolution diffusion-weighted imaging datasets. Hum Brain Mapp 32: 544–563. 20945352 10.1002/hbm.21043 PMC6870191

[21] Leuze CW, Dhital B, Anwander A, Pampel A, Heidemann R, et al (2011) Visualization of the orientational structure of the human stria of Gennari with high-resolution DWI. Proc Intl Soc Mag Reson Med 19: 2371.

[22] Leuze CWU, Anwander A, Bazin PL, Dhital B, Stüber C, et al (2014) Layer-specific intracortical connectivity revealed with diffusion MRI. Cereb Cortex 24: 328–339. 23099298 10.1093/cercor/bhs311 PMC3888365

[23] Leuze CWU, Anwander A, Geyer S, Bazin PL, Turner R (2012) High-resolution diffusion-weighted imaging of the orientational structure of motor and somatosensory cortex in human cadaver brain. Proc Intl Soc Mag Reson Med 20: 3594.

[24] Leuze CW, Bazin P, Anwander A, Dinse J, Wähnert M, et al (2012) Cortical profiles of diffusion weighted imaging (DWI) data differ between cortical areas. Proc Eur Soc Magn Reson Med Biol 29: 417.

[25] Kleinnijenhuis M, Sikma KJ, Barth M, Dederen P, Zerbi V, et al (2011) Validation of diffusion weighted imaging of cortical anisotropy by means of a histological stain for myelin. Proc Intl Soc Mag Reson Med 19: 2085.

[26] Kleinnijenhuis M, Zerbi V, Küsters B, Slump CH, Barth M, et al (2013) Layer-specific diffusion weighted imaging in human primary visual cortex in vitro. Cortex 49: 2569–2582. 23347559 10.1016/j.cortex.2012.11.015

[27] Liu C, Moseley ME, Bammer R (2005) Simultaneous phase correction and SENSE reconstruction for navigated multi-shot DWI with non-cartesian k-space sampling. Magn Reson Med 54: 1412–1422. 16276497 10.1002/mrm.20706

[28] Frank LR, Jung Y, Inati S, Tyszka JM, Wong EC (2010) High efficiency, low distortion 3D diffusion tensor imaging with variable density spiral fast spin echoes (3D DW VDS RARE). NeuroImage 49: 1510–1523. 19778618 10.1016/j.neuroimage.2009.09.010 PMC2791091

[29] Van AT, Hernando D, Sutton BP (2011) Motion-induced phase error estimation and correction in 3D diffusion tensor imaging. IEEE Trans Med Imaging 30: 1933–1940. 21652284 10.1109/TMI.2011.2158654

[30] Truong TK, Chen NK, Song AW (2012) Inherent correction of motion-induced phase errors in multishot spiral diffusion-weighted imaging. Magn Reson Med 68: 1255–1261. 22222689 10.1002/mrm.24124 PMC3323687

[31] Truong TK, Chen NK, Song AW (2011) Dynamic correction of artifacts due to susceptibility effects and time-varying eddy currents in diffusion tensor imaging. NeuroImage 57: 1343–1347. 21689763 10.1016/j.neuroimage.2011.06.008 PMC3138839

[32] Pruessmann KP, Weiger M, Börnert P, Boesinger P (2001) Advances in sensitivity encoding with arbitrary k-space trajectories. Magn Reson Med 46: 638–651. 11590639 10.1002/mrm.1241

[33] Jenkinson M, Bannister P, Brady JM, Smith SM (2002) Improved optimisation for the robust and accurate linear registration and motion correction of brain images. NeuroImage 17: 825–841. 12377157 10.1016/s1053-8119(02)91132-8

[34] Dale AM, Fischl B, Sereno MI (1999) Cortical surface-based analysis I: segmentation and surface reconstruction. NeuroImage 9: 179–194. 9931268 10.1006/nimg.1998.0395

[35] Anwander A, Pampel A, Knösche TR (2010) In vivo measurement of cortical anisotropy by diffusion-weighted imaging correlates with cortex type. Proc Intl Soc Mag Reson Med 18: 109.

[36] Ronen I, Ugurbil K, Kim DS (2005) How does DWI correlate with white matter structures? Magn Reson Med 54: 317–323. 16032693 10.1002/mrm.20542

[37] Budde MD, Annese J (2013) Quantification of anisotropy and fiber orientation in human brain histological sections. Front Integr Neurosci 7: 3. 23378830 10.3389/fnint.2013.00003 PMC3561729

